# Running-Related Overuse Injuries and Their Relationship with Run and Resistance Training Characteristics in Adult Recreational Runners: A Cross-Sectional Study

**DOI:** 10.3390/jfmk8030128

**Published:** 2023-09-05

**Authors:** Lea R. Stenerson, Bridget F. Melton, Helen W. Bland, Greg A. Ryan

**Affiliations:** 1 Department of Health and Human Performance, Concordia University of Chicago, River Forest, IL 60305, USA; bmelton@georgiasouthern.edu; 2 Department of Biology, Regis University, Denver, CO 80221, USA; 3 Department of Health Sciences and Kinesiology, Georgia Southern University, Statesboro, GA 30458, USA; 4 Department of Health Policy and Community Health, Georgia Southern University, Statesboro, GA 30458, USA; hwbland@georgiasouthern.edu; 5 Department of Health Sciences, Piedmont University, Demorest, GA 30535, USA

**Keywords:** running-related injury prevalence, recreational runners, resistance training, injury prevention

## Abstract

This study aimed to characterize running-related injuries (RRIs), explore their relationship with run and resistance training (RT) parameters, and identify perceived prevention measures among adult recreational runners. An anonymous online survey was designed and distributed via social media and email. Data were analyzed with chi-square, *t*-test, or analysis of variance (ANOVA), with significance accepted at *p* ≤ 0.05. Data from 616 participants (76.8% female, age: 42.3 ± 10.5 y) were analyzed. Most runners (84.4%) had an injury history, with 44.6% experiencing one in the past year. The most common RRI sites included the foot/ankle (30.9%) and knee (22.2%). RRI prevalence was higher in those running >19 miles weekly (48.4%, *p* = 0.05), but there were no differences based on RT participation status. Among those using RT, relatively more RRIs were observed in runners who trained the hip musculature (50.3%, *p* = 0.005) and did not include the upper body (61.6%, *p* < 0.001). A disproportionately high RRI prevalence was found for several of the other risk-reduction strategies. RRIs remain a substantial problem, particularly around the ankle/foot and knee. Higher run volume and performance motives were positively associated with RRIs. Most runners incorporated RRI risk-reduction techniques, with over half using RT. The current study did not determine whether preventative strategies were implemented before or after injury; therefore, prospective studies controlling for previous injuries are required to evaluate the effectiveness of RT in preventing future RRIs.

## 1. Introduction

Practical, efficient, and accessible, running is one of the most popular exercise modes worldwide, with involvement continuing to rise [1,2]. Along with higher participation rates, runner characteristics have evolved over the decades to include more female participants, a slower average pace, a higher average age [1], and those with health versus performance motives [1,3]. The current runner demographics exemplify the more casual, social, or recreational runner [4], who falls between novice and sub-elite or elite and ostensibly represents most of the running populace.

Recreational athletes may indeed reap the countless health benefits associated with running, including weight loss, cardiorespiratory fitness, lipoprotein profiles, mental health, and increased lifespan [5,6,7], but these rewards are concomitant with a high running-related injury (RRI) risk—defined herein as “running-related (training or competition) musculoskeletal pain in the lower limbs that causes a restriction or stoppage of running (distance, speed, duration, or training) for at least 7 days or 3 consecutive scheduled training sessions, or that requires the runners to consult a physician or other health professional” [8] (p. 375). RRIs are associated with direct and indirect costs (i.e., healthcare, time away from work) and represent a considerable economic burden [9,10]. Additionally, a history of an RRI is the main determinant of future RRIs and the primary reason people quit running [11,12]. Due to heterogeneous reporting methods and inconsistent definitions [8,13], RRI prevalence varies widely from 10 to 90%, with an average of 42.7% of runners experiencing an RRI annually [14,15]. Notably, these prevalence data were an amalgamation of novice to elite runners, triathletes, and orienteers and did not provide a unifying definition of RRI or delineate between the different athlete types, sex, age, or run distance.

The popularity of running, its indisputable benefits, and the high likelihood of nefarious outcomes highlight the necessity of incorporating RRI risk-reduction strategies. Efforts to reduce RRIs are not novel, but evidence of effective strategies remains elusive, likely due to the complexity of RRIs’ etiologies. Nonetheless, RRIs are universally characterized as a load–capacity imbalance [16], and while reducing RRIs requires a multifaceted approach, focusing on modifiable factors to improve runners’ capacity is imperative. Salient modifiable factors include strength and neuromuscular insufficiencies [17,18,19,20,21] and posture control or balance deficits [19,22,23]. Resistance training (RT), sometimes referred to as “weight” or “strength” training and described herein as requiring the body to resist an external force or load, can elicit positive neuromusculoskeletal adaptations, improving intrinsic capacity. For example, various RT modalities, from body weight to heavy load exercises, can correct strength imbalances; increase bone density; and improve overall strength, speed, power, balance, coordination, and posture control [24,25].

RT is posited to reduce injury prevalence in team sports [26]; however, the relationship between RT participation status and RRIs is equivocal among recreational runners. Two studies aimed to investigate the relationship between RT participation and RRIs in recreational runners [27,28], both reporting no benefit or association. However, Toresdahl et al. [27] did not account for RT participation in their observational group and reported poor compliance in the RT group. Voight et al. [28] found no association between RRIs and cross-training, but cycling was the most common cross-training modality, with RT representing only a small percentage. Moreover, no studies have investigated the specific RT programming parameters as they relate to RRIs, and little is known about the proportion of recreational runners who use RT to reduce RRIs or what other measures are perceived to achieve this goal. Thus, this study aimed to characterize overuse running-related injuries (RRIs), explore their relationship with specific run and resistance training (RT) parameters, and identify perceived prevention strategies among adult recreational runners. Uniquely, the current study: (a) used Yamato et al.’s [8] consensus definition of RRIs to assess overuse injuries, which are the most common RRI among distance runners [15]; (b) explicitly targeted recreational runners, defined as running an average of at least 2 times per week for at least a year, and considering running their primary exercise mode; (c) examined RRI’s association with RT participation and specific program parameters for all participants and by sex, age, and run distance; and (d) identified perceived prevention strategies currently in use.

## 2. Materials and Methods

### 2.1. Participants

Following institutional review board (IRB) approval, volunteers were recruited using a combination of non-probability purposeful convenience and snowball sampling. Inclusion criteria included recreational runners aged 18–65 who considered running their primary exercise mode and averaged at least 2 weekly runs for at least 1 year. Familiarity with the English language and internet access were requisite for study participation.

### 2.2. Procedures

This study used a quantitative, cross-sectional, online survey design. A 4-part survey was created with influence from related surveys [29,30,31,32,33,34,35,36,37,38] to reduce bias in question creation and promote consistency within the field. Each section (running history, RT characteristics, injury history, and standard demographics) had 2–11 questions, depending on the answers selected. The running-specific questions asked about years of experience, frequency, weekly distance, duration, and reasons for running. RT questions addressed participation status, experience, frequency, duration, workout parameters (i.e., sets, repetitions, effort level, type of RT, and targeted muscles), and reasons for participation. The RRI segment began with a definition of an overuse RRI that was adapted from other researchers [8]. Questions were asked about RRI history, the RRI prevalence in the past year, and the RRI location and severity if one was present. This section also assessed the use of perceived injury-prevention strategies.

The survey underwent unbiased peer review and was piloted with a small subset of the population for feedback and readability. A web-based Flesch–Kincaid readability test indicated a 7th–8th grade reading level, which is considered adequate for those 18 years and older. A brief study overview, an invitation to participate, and the Qualtrics (Provo, UT, USA) survey link were distributed broadly via Facebook (Menlo Park, CA, USA) and email lists with encouragement to share among other recreational runners. Survey questions were available only after agreeing to informed consent and eligibility criteria.

### 2.3. Statistical Analysis

All data were analyzed with IMB SPSS Statistics version 28 (Chicago, IL, USA). G*power’s (Aichach, Germany) minimum sample size for chi-square with a medium effect (Cohen’s W = 0.3), powered at 80%, and 5 degrees of freedom, was 143. Descriptive statistics are presented as mean and standard deviation (continuous variables) or frequency with percentage (categorical data). The survey questions yielded predominantly ordinal and nominal data. Cross-tabulation with chi-square analysis determined associations between the categorical variables. Independent *t*-tests or analysis of variance (ANOVA) were used for continuous data (e.g., years of experience). Significance was accepted at *p* ≤ 0.05 for all, and a post hoc Bonferroni correction was applied when omnibus significance was determined from the cross-tabulated chi-square analyses. Missing values were excluded from the analysis.

## 3. Results

### 3.1. Participants

Data from 616 eligible volunteers (76.8% female, M ± SD, age: 42.3 ± 10.5 y, body mass index (BMI) = 23.6 ± 3.6 kg∙m^−2^) were included in the analyses. On average, participants had about 13 years of experience and ran approximately four times per week, totaling 3–6 h. There were slight but statistically significant sex differences: men had a higher BMI and ran more frequently, while women had more running experience (Table 1).

### 3.2. Injury Prevalence and Characteristics

RRI prevalence for all runners and by sex, age, and run-distance categories are presented in Table 2. Nearly 85% of participants had a history of RRI, and about 45% reported one in the past year, with similar proportions across sex and age categories. RRI prevalence in the past year was higher than expected among those who ran >19 miles per week (48.4%), χ^2^(1) = 3.81, *p* = 0.05, and for those that selected “performance” as a dominant reason for running (51.3%), χ^2^(1) = 4.87, *p* = 0.03. Runners in the 51–65 age category were more likely than expected to experience an injury requiring moderate (vs. mild or major) training modifications (50%), χ^2^(1) = 10.86, *p* = 0.03.

**Figure 1:**
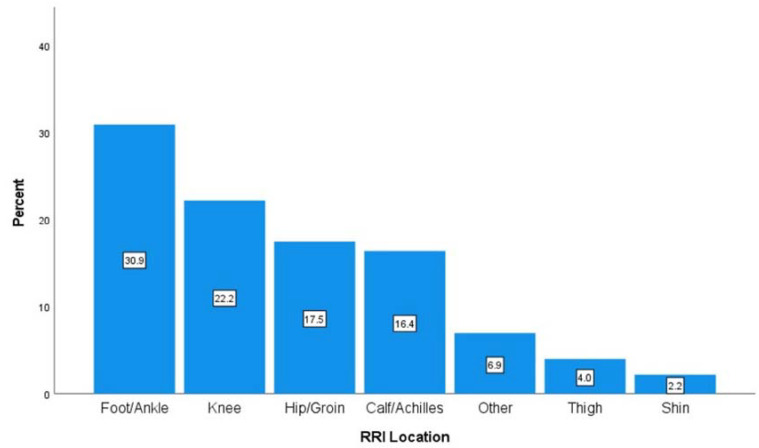
Percentage of injuries by anatomical location.

RRIs occurred most frequently at the foot/ankle (30.9%), knee (22.2%), hip/groin (17.5%), and calf/Achilles (16.4%), as presented in Figure 1. The proportion of RRIs at the calf/Achilles was higher than expected for men versus women (26.0% and 12.9%, respectively), χ^2^(6) = 14.32, *p* = 0.03. No other significant differences in injury location were determined across sex, age, and run-distance categories.

### 3.3. Relationships with Resistance Training Characteristics

No differences (*p* > 0.05) in RRI prevalence were observed between those who used RT and those who did not, which was consistent across sex, age, and run-distance categories (Table 3). Regarding RRI severity, sub-analysis showed that among those in the 35–50 age category who did not participate in RT, there was a lower proportion (14.3%) than expected of moderate RRI-related training modifications (*p* = 0.03).

A disproportionately high number of RRIs was observed in runners that included hip musculature in their RT (50.3%), χ^2^(1) = 7.97, *p* = 0.005, and in those that did not include the upper body musculature in their RT (61.6%), χ^2^(1) = 13.25, *p* < 0.001. Runners who selected “general health” as a reason for using RT were less likely than expected to have an RRI (42.1%), χ^2^(1) = 8.98, *p* = 0.003, while those using RT for performance gains were more likely to have an RRI (50.2%), χ^2^(1) = 4.23, *p* = 0.04. The 40.4% of runners following a personalized RT program—developed by an exercise professional such as a personal trainer, strength coach, or physical therapist—had a relatively higher RRI prevalence (52%), χ^2^(1) = 4.89, *p* = 0.03. Significant differences in RRI prevalence and severity were not observed (*p* > 0.05) across RT years of experience, duration of sessions, sets, repetitions, effort, and type of modality used (Table 4).

### 3.4. Strategies for Reducing Injury Risk

**Figure 2:**
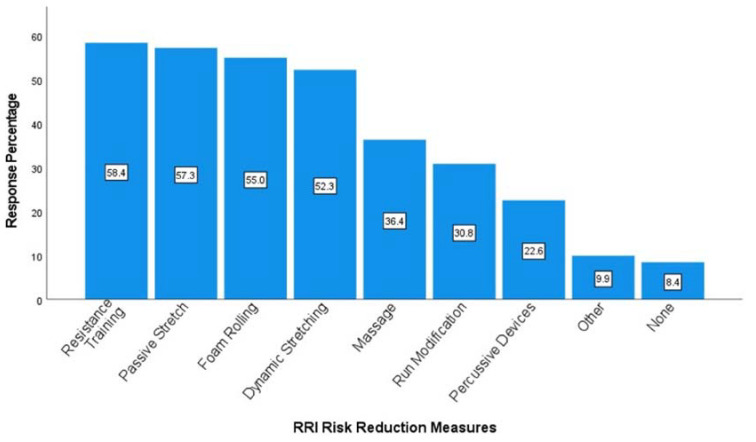
Percentage of runners using each strategy for reducing injuries (multi-response question).

Runners identified various strategies for injury prevention, with the most frequently reported methods including resistance training, passive stretching, foam rolling, and dynamic stretching (Figure 2). About 90% of all runners engaged with one or more strategies they perceived to help reduce injury risk. Runners using no risk-reduction measures had a disproportionately low RRI prevalence (21.2%), χ^2^(1) = 12.68, *p* < 0.001. There was a higher proportion of RRIs than expected among runners that used percussive devices (56.1%), χ^2^(1) = 9.56, *p* = 0.002, massage (51.3%), χ^2^(1) = 6.39, *p* = 0.01, dynamics (48.4%), χ^2^(1) = 3.95, *p* = 0.047, and who altered their run training (59.5%), χ^2^(1) = 24.45, *p* < 0.001. Sub-analyses revealed that those running >19 miles per week were more likely to use percussive devices (29.2%), χ^2^(1) = 16.79, *p* < 0.001, while those running fewer miles were more likely to include passive stretching (64.8%), χ^2^(1) = 13.13, *p* < 0.001. Males were also more likely to include passive stretching (67.8%), χ^2^(1) = 8.44, *p* = 0.004, and used run-training modifications more than expected (37.8%), χ^2^(1) = 4.18, *p* = 0.04. Whereas a higher proportion of females than males indicated using RT to reduce RRIs (60.7%), χ^2^(1) = 4.19, *p* = 0.04.

## 4. Discussion

The purpose of the current study was to characterize overuse RRIs, explore their relationship with specific run and RT parameters, and identify the perceived prevention strategies used by adult recreational runners. The investigation incorporated a consensus definition of RRIs with a focus on overuse injuries and is the first study to delineate RRI characteristics and the use of prevention methods, including RT and the specific RT parameters, across sex, age, and run-distance categories.

### 4.1. Injury Prevalence and Location

A major finding of our study is the alarmingly high likelihood of sustaining an RRI, with 85% of runners reporting a history of injury. Moreover, nearly half (44.6%) of the participants had incurred an injury within the past year. These results are consistent with other research with mixed populations that identified broad RRI prevalence ranges from about 19 to 80% [39] or 10 to 90%, averaging about 43% [14]. There were no differences in injury prevalence by sex, despite the finding that proportionally more males ran >19 miles per week, and there was a higher injury prevalence among those averaging greater distances (48.4%) versus those running ≤19 weekly miles (40.6%). This finding suggests that weekly running distance may be a more salient factor in RRIs than sex. Injury proportion by sex is somewhat mixed in the literature, with some reporting that females have a higher RRI risk relative to males [40] and others reporting the opposite [41,42]. There is supporting evidence that higher mileage is positively associated with RRIs [30,43]. Concomitantly, our results showed a higher proportion of RRIs than expected (51.3%) among recreational runners with event-performance motives, which aligns with studies reporting a high injury rate (67.4%) among competitive runners [30] and a positive relationship between running mileage and competition level [44].

The most common anatomical locations of RRIs were the foot/ankle and knee, which is consistent with findings from a recent systematic review [14], with the exception that in our study, foot/ankle injuries (30.9%) were more common than knee injuries (22.2%), rather than the reverse, as shown in the review. Earlier studies have also reported higher injury incidence at the knee (30.7%) compared to the ankle (8.3%) or foot (14.6%) [42]. As exemplified in our study, it is plausible that foot injuries may be on the rise, which, while still speculative, may be related to the growing popularity of carbon-plated super shoes [45].

### 4.2. Injury Associations with Resistance Training

No associations were found between RT participation status (yes or no) and RRI prevalence among all participants and within sex, age, and run-distance categories, nor were differences determined for RRI prevalence and severity across RT experience, session duration, sets, repetitions, effort, and type of modality used. Our overall prevalence results agree with other research also depicting no relationships between RT status and injuries [27,28]. Somewhat counterintuitively, a recent study of competitive runners determined that only a small percentage of those without RRIs had participated in RT activities [30], which aligns with the disproportionately high RRI prevalence revealed in the current study among those using hip muscles during RT. However, given the numerous beneficial neuromusculoskeletal adaptations that RT can stimulate [24,25,46] and the strong evidence of its efficacy in reducing injury risk in team sports [26,47,48], it is very likely that our and other’s [28] findings of no association or a positive association [30] between RT and RRI are the product of study design. Importantly, the current study design did not allow for a temporal determination of RT relative to sustaining an RRI; thus, it is unclear if RT use was preemptive or rehabilitative.

### 4.3. Strategies for Reducing Injury Risk

About 10% of runners in this study do not use RRI-prevention strategies, while other studies have found that nearly 20% do not use injury-reduction strategies [31]. RT, passive stretching, foam rolling, and dynamic stretching were most frequently used to reduce injury risk, with each selected by just over 50% of runners. Many competitive runners also use RT, stretching, and foam rolling (62.5%, 86.2%, and 54.7%, respectively) [30]. Interestingly, the current study found that several of the perceived prevention strategies were associated with a high proportion of RRIs. However, as with RT participation, the time frame for commencing injury-prevention measures was not determined, and sustaining an injury is likely to facilitate the incorporation of risk-reducing strategies [31]. Nonetheless, understanding prevention preferences for the sub-populations (sex, age, and distance categories) can inform exercise and healthcare professionals about preferences among these runners—with the caveat that conclusive evidence about the efficacy of each strategy for reducing RRIs is scarce.

### 4.4. Limitations

Important limitations exist for this study aside from the design not allowing for the elucidation of the timeline for RRIs and RT use or other prevention measures, thus precluding causal inferences. The study results were subject to recall bias as the survey was self-administered and self-reported. Survey questions were predominantly closed-ended, and more nuanced responses may have been generated by including open-ended questions. Though inclusion criteria were intentionally broad to approximate the larger population of recreational runners, convenience sampling led to a disproportionally high percentage of female, well-educated, and Caucasian runners, thus reducing external validity and limiting applicability to the current study population. However, considering the traditionally male-dominated nature of research, the high representation of females in this study is simultaneously a strength.

## 5. Conclusions

Recreational runners’ risk of RRI is high, with an overall prevalence of about 85% and an annual prevalence of nearly 50%. While completely eradicating RRIs is unrealistic, coaches and practitioners should educate recreational runners about the high RRI prevalence and encourage proactive risk-reducing measures, particularly for those running higher distances and with performance motives. Injury-prevention measures, including RT, were not associated with lower RRIs in this study, but these results were substantially confounded by participation timing considerations, which were not determined herein. Nonetheless, RT can improve runners’ capacity to tolerate training load and, thus, should be recommended. Lastly, cross-sectional retrospective studies are not adequate to elucidate the effect of RT on reducing future RRIs—prospective studies that control for previous injuries while tracking the use and timing of RT and other prevention measures relative to the RRI are necessary.

## Figures and Tables

**Table 1:** Participant characteristics.

Variable	All	Female	Male
Total	616 (100%)	473 (76.8%)	143 (23.2%)
Age (y)	42.3 ± 10.5	42.3 ± 10.1	42.3 ± 11.8
BMI (kg∙m^−2^)	23.6 ± 3.6	23.3 ± 3.7	24.9 ± 3.2 ***
Education			
High school or equivalent	29 (4.7%)	14 (3%)	15 (10.5%) **
Trade/technical	20 (3.2%)	15 (3.2%)	5 (3.5%)
Associates	22 (3.6%)	14 (3%)	8 (5.6%)
Bachelors	202 (32.8%)	155 (32.8%)	47 (32.9%)
Masters/doctorate	342 (55.5%)	275 (58.1%)	67 (46.9%)
Community			
Urban	130 (21.1%)	97 (20.5%)	33 (23.1%)
Suburban	374 (60.7%)	295 (62.4%)	79 (55.2%)
Rural	111 (18%)	81 (17.1%)	30 (21%)
Race			
Asian/Pacific Islander	12 (1.9%)	7 (1.5%)	5 (3.5%)
Black/African American	5 (0.8%)	5 (1.1%)	-
Native American/Alaskan	1 (0.2%)	-	1 (0.7%)
White/Caucasian	565 (91.7%)	436 (92.2%)	129 (90.2%)
Bi- or multi-racial	13 (2.1%)	10 (2.1%)	3 (2.1%)
Other	19 (3.1%)	14 (3.0%)	5 (3.5%)
Run experience (y)	12.8 ± 9.6	13.3 ± 9.6 *	11.3 ± 9.7
Frequency (d/wk)	3.95 ± 1.3	3.9 ± 1.2	4.3 ± 1.4 **
Weekly distance (miles)			
≤19	298 (48.4%)	242 (51.2%)	56 (39.2%)
>19	318 (51.6%)	231 (48.8%)	87 (60.8%) *
Weekly duration (h)			
1–2	79 (12.8%)	60 (12.7%)	19 (13.3%)
3–4	22 8 (37%)	186 (39.3%)	42 (29.4%)
5–6	18 1 (29.4%)	136 (28.8%)	45 (31.5%)
7+	128 (20.8%)	91 (19.2%)	37 (25.9%)

*Note*. Continuous data are presented as *M* ± *SD.* Categorical data are presented as frequency (*n*) and percentage. BMI = body mass index. * *p* < 0.05, ** *p* < 0.01, *** *p* < 0.001.

**Table 2:** Injury characteristics by frequency and percentage.

Variable	Category (*n*)	History of RRI	RRI in the Past Year
	Total (*n* = 616)	520 (84.4%)	275 (44.6%)
Sex	F (*n* = 473)	398 (84.1%)	202 (42.7%)
	M (*n* = 143)	122 (85.3%)	73 (51.0%)
*p*		0.74	0.08
Age	18–34 (*n* = 144)	120 (83.3%)	71 (49.3%)
	35–50 (*n* = 327)	277 (84.7%)	140 (42.8%)
	51–65 (*n* = 145)	123 (84.8%)	64 (44.1%)
*p*		0.92	0.42
Run (miles/wk)	<19 (*n* = 298)	250 (83.9%)	121 (40.6%)
	19+ (*n* = 318)	270 (84.9%)	154 (48.4%) *
*p*		0.73	0.05

*Note*. RRI = running-related injury. F = female, M = male. * *p* ≤ 0.05.

**Table 3:** Running-related injuries and resistance-training status across sex, age, and run-distance categories.

	RRI in Past Year		*p*	RRI Severity			*p*
	Yes	No		Mild	Moderate	Major	
All			0.49				0.13
Yes	195 (45.6%)	233 (54.4%)		57 (29.2%)	73 (37.4%)	65 (33.3%)	
No	80 (42.6%)	108 (57.4%)		30 (37.5%)	20 (25.0%)	30 (37.5%)	
Sex							
Female			0.08				0.37
Yes	156 (45.1%)	190 (54.9%)		47 (30.1%)	61 (39.1%)	48 (30.8%)	
No	46 (36.2%)	81 (63.8%)		15 (32.6%)	13 (28.3%)	18 (39.1%)	
Male			0.33				0.24
Yes	39 (47.6%)	43 (52.4%)		10 (25.6%)	12 (30.8%)	17 (43.6%)	
No	34 (55.7%)	27 (44.3%)		15 (44.1%)	7 (20.6%)	12 (35.3%)	
Age							
18–34							
Yes	51 (50.0%)	51 (50.0%)	0.80	14 (27.5%)	14 (27.5%)	23 (45.1%)	0.33
No	20 (47.6%)	22 (52.4%)		9 (45.0%)	5 (25.0%)	6 (30.0%)	
35–50							
Yes	98 (43.2%)	129 (56.8%)	0.84	32 (32.7%)	36 (36.7%)	30 (30.6%)	0.03
No	42 (42.0%)	58 (58.0%)		18 (42.9%)	6 (14.3%) *	18 (42.9%)	
51–65							
Yes	46 (46.5%)	53 (53.5%)	0.41	11 (23.9%)	23 (50.0%)	12 (26.1%)	0.76
No	18 (39.1%)	28 (60.9%)		3 (16.7%)	9 (50.0%)	6 (33.3%)	
Run Distance (miles)							
≤19							
Yes	89 (41.8%)	124 (58.2%)	0.51	31 (34.8%)	36 (40.4%)	22 (24.7%)	0.06
No	32 (37.6%)	53 (62.4%)		9 (28.1%)	8 (25.0%)	15 (46.9%)	
>19 miles							
Yes	106 (57.3%)	79 (42.7%)	0.65	26 (24.5%)	37 (34.9%)	43 (40.6%)	0.06
No	48 (49.3%)	55 (53.4%)		21 (43.8%)	12 (25.0%)	15 (31.3%)	

*Note*. RT = resistance training. RRI = running-related injury. RRI severity reflects the extent to which training was altered. Data are presented as frequency (*n*) and percentage. In the case of omnibus significance, a post hoc Bonferroni adjustment was applied. * *p* < 0.001 after Bonferroni adjustment.

**Table 4:** Resistance-training characteristics by running-related injury status.

Characteristics	RRI in Past Year		*p*
	Yes (*n* = 195)	No (*n* = 233)	
RT experience (y)	8.6 ± 9.1	9.5 ± 9.0	0.32
RT frequency (d/wk)	2.6 ± 1.2	2.5 ± 1.1	0.44
RT min/session	30–44 (72, 44.7%)	30–44 (89, 55.3%)	0.62
Repetition range	7–12 (128, 46.4%)	7–12 (148, 53.6%)	0.99
Effort level (0–10)	6.2 ± 1.4	6.2 ± 1.4	0.66

*Note*. RT = resistance training. RRI = running-related injury. Data are presented as *M* ± *SD* or as mode with frequency (*n*) and percentage.

## Data Availability

Data available upon reasonable request.
